# New York County

**Published:** 1899-06

**Authors:** Robert W. Ellis

**Affiliations:** Secretary


### VETERINARY MEDICAL ASSOCIATION OF NEW YORK COUNTY.

The regular monthly meeting of the Veterinary Medical Association of New York County was called to order by President Robertson, at the New York Academy of Medicine, 17 West Forty-third Street, on May 3, at 8 P.M.

The following members were present at roll-call: Drs. Bell, Clayton, Dickson, Ellis, Foy, Gill, Goubeaud, Keller, MacKellar, O'Shea, and Robertson. The following gentlemen were present as visitors: Drs. L. Nicolas, N. Reichman, and G. B. Morse.

Dr. Dickson read a very interesting paper on "Azoturia in the Dog,"[1] which was freely discussed.

Dr. Bell: I was very anxious to hear Dr. Dickson's paper on "Azoturia in the Dog," as a short time ago, in answering an inquiry from Dr. Dickson in relation to this condition, I stated that I had had a case that day. The symptoms observed by me, however, were more like those described by Dr. Leech than those observed by Dr. Dickson. My case was that of a fox-terrier, abnormally fat; had eaten breakfast, felt good, ran out, and suddenly became paralyzed. He sat up in front, but could not use hind legs. I gave calomel, produced catharsis, and the case terminated favorably. I have had another case since in a daschshund; symptoms similar; treatment the same; recovery. If it is not azoturia, what is it?

Dr. Dickson: I have often had cases in which they would drag the hind legs for two or three weeks, but yield to treatment; but they were somehow different from the cases described in my paper.

Dr. Bell: Did you make an analysis of the urine?

Dr. Dickson: No.

Dr. Robertson: Did you observe any appearance of jaundice?

Dr. Dickson: Yes, a slight trace of it in the second dog; I did not look close enough at the first one to observe it.

Dr. Robertson: The discoloration on this blotter from the sample of urine you furnished us looks as though it might be due to bile.

Dr. Gill: One or two points in Dr. Dickson's paper bear out his diagnosis, viz., the hardness and swelling of the gluteal muscles, with characteristic appearance of urine, and the fact that it occurred in plethoric animals, with lack of exercise, etc. I think the coloring-matter in this sample of urine resembles the hæmoglobinuria rather than bile, and with Dr. Dickson's consent I will have it tested at the Board of Health Laboratory, and report the result of the test at the next meeting.

Dr. Goubeaud reported a case similar to Dr. Dickson's in a fox-terrier let out to urinate; came in stiff and trembling; muscles hard, and could not stand; was put on strychnine and calomel; died; post-mortem revealed nothing wrong with intestinal tract.

Dr. Gill gave a discourse on "Some Important Veterinary Events of Next September." This subject was fully discussed.

[1] See page 376.

Moved and seconded that Drs. Dickson and Gill receive a vote of thanks for their papers; carried.

Judiciary Committee reported progress.

Moved by Dr. Clayton, that the Secretary be authorized to issue certificates to members who are shown to be square on the books, and pay an additional dollar to cover expenses of engrossing, mailing, etc., instead of $5, as heretofore; seconded and carried. The Secretary was so authorized by the President.

ROBERT W. ELLIS, D.V.S.,
Secretary.

